# Predictive value of C-Reactive Protein/Albumin ratio in patients with chronic complicated diabetes mellitus

**DOI:** 10.12669/pjms.35.6.618

**Published:** 2019

**Authors:** Muharrem Bayrak

**Affiliations:** Muharrem Bayrak, MD. Assistant Professor, Department of Internal Medicine, Regional Training and Research Hospital, Erzurum, Turkey

**Keywords:** Albumin, Complications, C-reactive protein, Diabetes mellitus

## Abstract

**Objective:**

To investigate the relationship between serum C-reactive protein (CRP)/albumin (ALB) ratio and complication occurrence in patients with Type-II diabetes mellitus with at least one chronic complication.

**Methods:**

The CAR, demographic characteristics, and other parameters of 108 patients with at least one chronic diabetic complication who attended to the internal medicine outpatient clinic between January 1, 2017, and September 1, 2018, were retrospectively evaluated. Healthy control subjects who did not have any systemic or infectious diseases were also included in the study. I compared the CAR, demographics, and other blood parameters between the two groups were compared.

**Results:**

The mean CAR levels were significantly higher in diabetic patients with at least one complication compared to the control group (0.15 [0.07 - 0.29] vs 0.07 [0.07 - 0.07], respectively, p<0.001). There was no significant correlation between CAR and diabetic complications, including neuropathy, nephropathy, coronary artery disease, and retinopathy in the patient group (p>0.05 for all). In the receiver operating curve (ROC) analysis, there was no significant cut-off point for CAR predicting diabetic complications.

**Conclusions:**

Although serum CAR levels were significantly higher in complicated diabetic patients compared to controls, any validated CAR value for predicting diabetic complications were not observed.

## INTRODUCTION

Diabetes mellitus (DM) is a heterogeneous metabolic disease which affects millions of people worldwide. The prevalence of DM is expected to be double by 2030.^1^ Uncontrolled hyperglycemia in DM may cause acute and long term complications, which cause increased morbidity and mortality. The most common long term complications of DM are microangiopathy, i.e., retinopathy, neuropathy, nephropathy, and macroangiopathy, i.e., coronary artery disease.^2^

Inflammation is significantly correlated with diverse clinical conditions associated with characteristic changes in the serum acute-phase proteins. C-reactive protein (CRP) and albumin are well-known acute-phase reactants (APR) and have been used as critical inflammatory biomarkers for predicting morbidity and mortality in many diseases.^3^–^6^ CRP levels increase (positive APR) whereas albumin levels decrease (negative APR) during inflammation. However, serum levels of these mediators may be influenced by several factors, including age, body mass index (BMI), insulin resistance, ethnicity, estrogen use, and nutritional status.

CRP to albumin ratio (CAR), a newly introduced indicator, is believed to be a more reliable predictor of the inflammatory status than CRP or albumin alone. The CAR has been used in the evaluation of prognosis and mortality in many diseases, including malignancies as a prognostic score.^7^

In the present study the relationship between the CAR and complication occurrence in patients with Type-II DM was investigated. The predictive value of the CAR for the complications of DM is also evaluated.

## METHODS

### Study Population

A total of 108 consecutive patients with Type-II DM and 108 healthy controls who attended to internal medicine outpatient clinic between 1 January 2017 and 1 September 2018 were included in this retrospective observational study. This study was conducted in accordance with the Declaration of Helsinki. The local institutional ethics committee approved the study.

Age, sex, duration of DM, history of smoking, blood and urine biochemical analyses including HbA1c, total cholesterol, low-density lipoprotein cholesterol (LDL-C), high-density lipoprotein cholesterol (HDL-C), triglycerides (TG), fasting glucose, postprandial glucose, erythrocyte sedimentation rate (ESR), white blood cell count (WBC), CRP, albumin, CAR, glomerular filtration rate (GFR), proteinuria, and creatinine were retrospectively reviewed from electronic file system and file records of the participants.

Complications of Type-II DM were determined using biochemical analyses, coronary angiography, electromyography (EMG), and dilated fundus examination in consultation with nephrology, cardiology, neurology, and ophthalmology departments. Patients with at least one chronic complication of DM were included in the study.

### Demographic Measurements

Body mass index (BMI) was calculated for all participants as body weight/height^2^ (kg/m^2^).

### Exclusion criteria

Patients with active infectious disease, chronic inflammatory or autoimmune disease, cerebrovascular disease, morbid obesity, peripheral arterial disease, hematological diseases, end-stage hepatic and renal disease, malnutrition, and malignancy were excluded from the current study.

### Biochemical analysis

Venous blood samples were obtained from antecubital veins after an overnight fast. Hemoglobin, glucose, creatinine, albumin, CRP, LDL-C, HDL-C, and TG levels were analyzed in the laboratory of our institution. GFR levels were measured with Modification of Diet in Renal Disease (MDRD) formula.^8^ For our institution, while the CRP levels ranged from 0-5 mg/dl, the albumin level ranged from 3.5-5.2 mg/dl. The CAR value is determined by dividing the CRP level with the albumin level.

### Statistical analysis

Statistical analyses were performed by R 3.5.1 for Windows using sm and WRS2 packages (R Foundation for Statistical Computing, Vienna, Austria), Jamovi Computer Software (Version 0.9, retrieved from https://www.jamovi.org), and MedCalc Statistical Software Trial version. Descriptive statistics were presented as mean and standard deviation or median (IQR) for continuous variables and as frequency with percentage for categorical variables. The Kolmogorov-Smirnov test was used to evaluate the normality test for the numeric variables. Independent Samples t-test was used to examine the differences between the normally distributed numerical variables of the groups. Since the mean age is significantly different between the groups, the age variable was controlled as a covariate with ANCOVA models. Differences between categorical variables were performed by Pearson Chi-Square Test in 2x2 tables and Fisher’s Exact Test in RxC tables. Spearman Rho correlation coefficient was used to examine the relationships between numerical variables. Receiver operating characteristic (ROC) curves and area under the curve (AUC) were used to define the best cut-off values of CAR, CRP, and albumin for predicting chronic DM complications. A p-value less than 0.05 was accepted as statistically significant.

### Power analysis

Since there was no similar study in the literature, a preliminary study was conducted with 10 individuals in order to determine the effect size (amount of difference between groups) and to perform power analysis. Only descriptive statistics for CAR were calculated from this study, and the effect size was estimated. The statistical significance of a difference of 0.040 units in terms of CAR between the groups under 80% power and 5% type I error conditions (α=0.05), it was determined that at least 82 individuals in each group should be included with a total of 164 individuals. On the other hand, when the power of the study was calculated from the data obtained from 108 patients and 108 control group individuals, it was concluded that it was 95% (Post Hoc Power Analysis). Power analysis was performed with G * Power 3.1.9.4 for Windows (Open Source).

## RESULTS

A total of 108 patients with Type-2 DM in the present study, 48 (44.44%) were male and 60 (55.56%) were female. In the control group, 48 (44.44%) were male and 60 (55.56%) were female. The mean age of the patient and control groups were 56.87 ± 11.55 years and 37.02 ± 11.56 years, respectively. The duration of DM was 12.56 ± 3.44 years. The shortest duration of DM was seven years and the longest one was 24 years. The mean HbA1c (%) levels of the patient and control groups were 8.9 ± 2.3 and 5.6 ± 0.3, respectively. There was a statistically significant difference between the groups in terms of age (p<0.001). Therefore, age-corrected covariance analysis was performed to eliminate age-related mixing effects on the results. The mean CAR values of the patient and control groups were 0.15 (0.07 - 0.29) and 0.07 (0.07 - 0.07), respectively (p<0.001). The clinical and biochemical parameters of the groups are summarized in Table-I in detail.

**Table I:** The demographic, clinical and biochemical parameters of the study population.

	Group		Covariate (p)	

	Patient (n=108)	Control (n=108)	p	Age
Age	56.87 ± 11.55	37.02 ± 11.56	<0.001*	-
Gender (Male / Female)	48 (44.44) / 60 (55.56)	48 (44.44) / 60 (55.56)	1.000***	-
Duration of diabetes	12.56 ± 3.44	-	-	-
Smoking	51 (47.22)	28 (25.93)	0.001***	-
HbA1c (%)	9.00 ± 2.34	5.61 ± 0.30	<0.001	0.173**
Total Cholesterol, mg/dl	200 [169.5 - 233]	184.5 [162 - 206.5]	0.005	0.024****
LDL-C, mg/dl	122.88 ± 35.76	114.73 ± 29.53	0.069	
HDL-C, mg/dl	43.47 ± 10.6	50.69 ± 14.22	<0.001	0.859****
Triglycerides, mg/dl	153.5 [109 - 239]	100.5 [65 - 155]	<0.001	<0.001****
BMI(kg/m2)	27.52 ± 4.45	26.72 ± 4.7	0.203	
Fasting Glucose, mg/dl	162.1 ± 62.34	79.82 ± 6.27	<0.001	<0.001**
Postprandial Glucose, mg/dl	232.5 [202 - 286]	110 [93.5 - 130]	<0.001	<0.001****
ESR	11 [5 - 18.5]	9 [7 - 11]	0.163	
WBC (10^9^/L)	7913.62 ± 2081.68	8046.92 ± 546.06	0.522	
CRP, mg/dl	0.62 [0.32 - 1.25]	0.3 [0.3 - 0.3]	<0.001	<0.001****
Albumin, mg/dl	4.32 [4.15 - 4.51]	4.46 [4.25 - 4.58]	0.003	0.971****
CAR	0.15 [0.07 - 0.29]	0.07 [0.07 - 0.07]	<0.001	<0.001****
GFR(MDRD)	92 [77 - 99]	107.5 [97 - 117]	<0.001	0.152****
Proteinuria, mg/day	143.5 [251.5 – 83.5]	8 [9 - 7]	<0.001	<0.001****
Creatinin, mg/dl	0.84 [0.72 - 1.07]	0.74 [0.67 - 0.8]	<0.001	0.330****
***Precence of complications***				
Retinopathy	21(%19.44)	0 (0)	-	-
Neuropathy	102(%94.44)	0 (0)	-	-
Nephropathy	51(%47.22)	0 (0)	-	-
CAD	22(%20.37)	0 (0)	-	-

Bold values are significant at p < 0.05, LDL: Low-density lipoprotein, HDL: High-density lipoprotein, BMI: Body mass index, ESR: Erythrocyte sedimentation rate, WBC: White blood cell, CRP: C-reactive protein, CAR: C-reactive protein albumin ratio, GFR: Glomerular filtration ratio, MDRD: Modification of diet in renal disease; CAD: Coronary artery disease,

* The independent samples t-test was used,

** The groups’ means were adjusted for age by using parametric covariance analysis (ANCOVA),

*** Chi-square test was used,

**** The groups’ means were adjusted for age by using non-parametric covariance analysis (sm. ANCOVA).

No correlation between CAR and age, duration of diabetes, HbA1c, total cholesterol, HDL-C, TG, BMI, fasting glucose, postprandial glucose, WBC, albumin, GFR, proteinuria, and creatinine levels (p> 0.05 for all) was observed. The CAR values were significantly correlated with LDL-C (r = 0.227, p = 0.018), ESR (r = 0.449, p<0.001), and CRP (r = 0.986, p<0.001). There was no significant correlation between the CAR and diabetic complications, including neuropathy, nephropathy, coronary artery disease, and retinopathy in the patient group (p>0.05 for all). Table-II shows the correlation analysis in detail.

**Table II:** The correlation between C-reactive protein-albumin ratio and other parameters in diabetic patients.

	r	p
Age	0.022	0.823
Duration of diabetes	-0,033	0,736
Hba1c	0.107	0.270
Total Cholesterol, mg/dl	0.156	0.107
LDL-C, mg/dl	0.227	0.018
HDL-C, mg/dl	0.029	0.769
Triglycerides, mg/dl	0.059	0.545
BMI(kg/m2)	-0.058	0.552
Fasting Glucose, mg/dl	0.143	0.140
Postprandial Glucose, mg/dl	0.138	0.155
ESR	0.449	< 0.001
WBC (10^9^/L)	0.065	0.506
CRP, mg/dl	0.986	< 0.001
Albumin, mg/dl	-0.162	0.094
GFR(MDRD)	0.041	0.677
Proteinuria, mg/day	0.158	0.102
Creatinine, mg/dl	-0.090	0.353

The spearman rho correlation coefficient was used. Bold p values are significant at p < 0.05, LDL: Low-density lipoprotein, HDL: High-density lipoprotein, BMI: Body mass index, ESR: Erythrocyte sedimentation rate, WBC: White blood cell, CRP: C-reactive protein, GFR: Glomerular filtration ratio, MDRD: Modification of diet in renal disease.

**Fig. 1:**
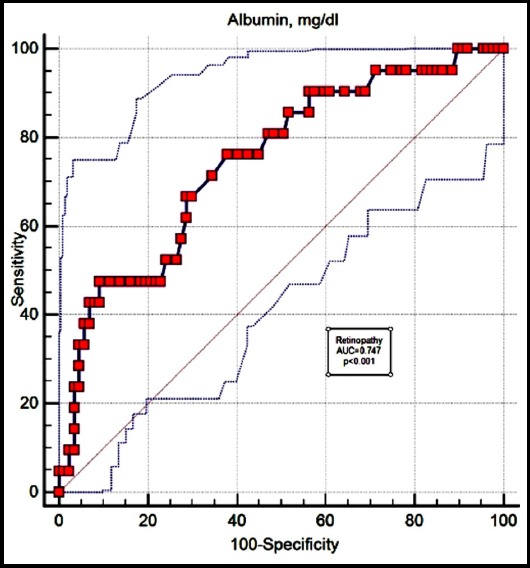
The receiver operating characteristic (ROC) curve of serum albumin in the prediction of retinopathy from diabetes mellitus.

**Fig. 2:**
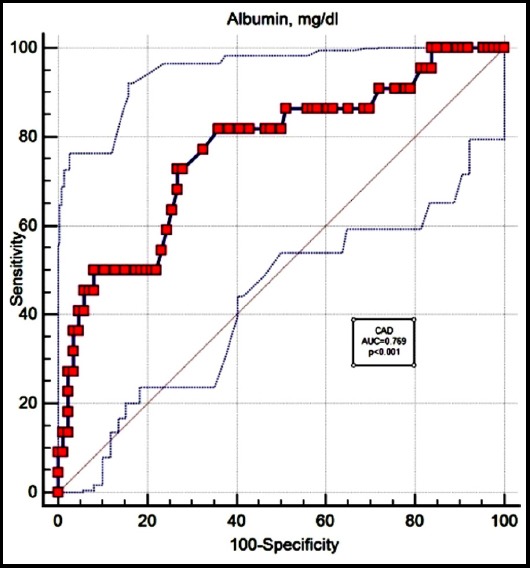
The receiver operating characteristic (ROC) curve of serum albumin in the prediction of coronary artery disease from diabetes mellitus.

ROC analyses have shown that there is no optimum CAR or CRP value cut-off point for predicting diabetic complications. However, serum albumin cut-off points for predicting retinopathy and coronary artery disease complications were significant (Fig. 1 and 2, respectively). The cut-off value of albumin assessed by ROC curve is 4.02 mg/dl with 47.62% sensitivity and 90.80% specificity (area under curve 0.747) for predicting retinopathy. The cut-off value of albumin assessed by ROC curve is 4.26 mg/dl with 72.73% sensitivity and 73.26% specificity (area under curve 0.769) for predicting CAD.

## DISCUSSION

Diabetes mellitus is a chronic systemic inflammatory disease of glucose metabolism with increasing complications every year.^9^ DM leads to many complications, most of which is associated with the duration of illness. These complications cause increased morbidity and mortality rates and high economic burden.^10^ Therefore, predicting those complications is of critical importance. In the present study, it was shown that CAR levels of complicated diabetic patients were higher than those of healthy controls. It was also demonstrated that no validate CAR cut-off level is present for predicting diabetic complications.

CRP is a well-known predictive marker for myocardial infarction and stroke in healthy subjects. It has also been shown that CRP levels are correlated with the severity of CAD, recurrent coronary events, and mortality in patients with stable CAD.^11^ Additionally; CRP levels have been assessed to be a predictive marker for CAD in hypertension. Serum CRP levels are clinically used to predict the existence of cardiovascular events.^12^ Many clinical trials have investigated the relevant predictive biomarkers value of circulating CRP levels for patients with DM.^13^-^15^ Wanget al.^13^ assessed the association between CRP values and Type-II DM risk by conducting a meta-analysis. This meta-analysis has revealed that elevated levels of CRP in healthy subjects are significantly associated with high-risk in Type-II DM.^13^ It has also been shown that CRP values could be used to predict DM complications in a recent study assessing the relationship between inflammatory markers and endothelial dysfunction in patients with DM.^14^ The authors also reported that well-controlled metabolic features in DM might reduce the CRP levels.^14^ Roy et al.^15^ said that increased CRP levels were independent predictors of the incidence of cardiovascular disease in patients with DM. In the present study, a higher CRP levels in patients with Type-II DM who had at least one chronic complication compared to controls was observed. However, it was also found that there was no predictive value of serum CRP levels for the complications of DM.

Albumin is the major plasma protein, constituting about 60% of the total serum proteins. Serum albumin level is a remarkably strong prognostic indicator of morbidity and mortality in many diseases. Although CRP is positively correlated with the acute-phase reaction, there is an inverse correlation between albumin and acute-phase reaction. Serum albumin levels are decreased in chronic inflammatory conditions.^16^ Several studies have highlighted that the lower concentrations of albumin in serum are associated with an increased risk of CAD, cardiovascular mortality, and carotid atherosclerosis.^17^,^18^ Bae et al.^19^ found increased serum albumin level to be associated with insulin resistance. However, the authors did not find the serum albumin as an independent effect on the development of diabetes. In the present study, a significant difference between the patient and control groups in terms of serum albumin levels is observed. However, age-corrected covariance analysis showed no significant difference between the groups. ROC analysis in diabetic patients showed albumin had a predictive value for retinopathy and CAD complications in this cohort.

The CAR reflects the balance between CRP and albumin levels and has prognostic significance in a variety of inflammatory disorders including sepsis and malignancies.^7^ The higher the CAR value is, the worse the prognosis is and the greater the inflammatory burden is. Recently, CAR has become an area of interest for many inflammatory disorders.^20^–^22^ Wei et al.^23^ have shown that increased CAR values were independently related to increased 30-day mortality risk in critically ill patients. Similarly, Park et al.^24^ have assessed the role of the CAR values for critical care patients, and they have shown that CAR values are associated with increased 28-day mortality risk in critically ill patients. The authors have also speculated that CAR values could be used as a predictor of 28-day mortality. In a previous study, CAR and CRP values were assessed in CAD. The authors reported higher CAR values to be independently related to the severity, complexity, and extent of CAD.^25^ In the present study, I found that the CAR levels were significantly higher in complicated diabetic patients compared to healthy controls. However, there is no significant validate CAR cut-off point for predicting diabetic complications. Therefore, the CAR values could not be attributed as a predictive value or marker role for anticipating diabetic complications.

### Limitations of the study

This study has several limitations. The relatively small sample size of patients with Type-II DM and the retrospective nature of the study are the main limitations. Since the study was performed in a single-center, the number of patients was small to achieve a sub-group analysis. Several confounding conditions, including nutritional status, comorbidities, and undocumented medications that we cannot manage entirely may have influenced the levels of serum CRP and albumin.

## CONCLUSIONS

The study found that serum CAR levels were significantly higher in complicated diabetic patients compared to controls. However, I did not observe any validate CAR cut-off value for predicting diabetic complications were not observed. Future studies with larger sample size are needed to clarify the relationship between the CAR levels and chronic complications of diabetes.
